# 

**Published:** 1897-09

**Authors:** 


					CONTENTS.
PAGE I
Fifteen Years' Experience of Infec-
tious Diseases in a Public School.
By W. JOHNSTONE Jr YFFE, 15. A.,
M.D. Dub
221
The Causes of Death in Five Cases
in a Colliery Explosion. By
Trafford Mitchell, M.JD. Glasg.,
D.P.H. Camb
235
Two Cases of Intubation in Adults.
By Harold F. Mole, F.R.C.S. Eng.
237
A Case of Haemophilia with Joint
Lesions.
By J. E. Shaw, M.B. Ed
240
Progress of the Medical sciences ?
Medicine.
J. Michell Clarke,
246
Surgery.
W. H. HARSANT
251
Dermatology.
Henry Waldo, M.D.
254 :
Reviews of Books
260
Notes on Preparations for the Sick
286
The Library of the Bristol Medico-
Chirurgical Society 289
Local Medical Notes
292
Scraps
299

				

